# Genetic variations in the sheep *SIRT7* gene and their correlation with body size traits

**DOI:** 10.5194/aab-62-189-2019

**Published:** 2019-04-16

**Authors:** Hongwei Xu, Xiaoyu Zhang, Rongxin Zang, Yong Cai, Xin Cao, Jutian Yang, Jie Li, Xianyong Lan, Jianping Wu

**Affiliations:** 1 College of Animal Science and Technology, Gansu Agricultural University, Lanzhou, Gansu, China; 2 Science Experimental Center, Northwest Minzu University, Lanzhou, Gansu, China; 3 College of Life Science and Engineering, Northwest Minzu University, Lanzhou, Gansu, China; 4 College of Animal Science and Technology, Northwest A&F University, Yangling, Shaanxi, China; a current address: College of Animal Science and Technology, Gansu Agricultural University, Lanzhou, Gansu, China

## Abstract

As a nicotinamide adenine dinucleotide (NAD)-dependent histone deacetylase and ADP ribosyl transferase, the silent information regulator 7 (Sirtuin 7, SIRT7) plays a crucial role in regulating the differentiation of adipocytes and myoblasts, lipid metabolism, glucose metabolism, and cellular growth in mammals. It has been hypothesized that SIRT7 affects growth traits in animals; therefore, in this study, the potential insertion/deletion (indel) of genetic variations within the ovine *SIRT7* gene and their correlation with sheep growth traits were explored. A total of 709 individuals from five Chinese and Mongolian sheep breeds were analyzed. Two novel indel loci of the sheep *SIRT7* gene were detected and were named 5${}^{\prime }$ promoter region-insertion-7 bp (5${}^{\prime }$ promoter region-7 bp) and 3${}^{\prime }$ UTR-insertion-17 bp (3${}^{\prime }$ UTR-17 bp), respectively. In all of the sheep breeds, frequencies of the 5${}^{\prime }$ promoter region-7 bp mutation were low, whereas mutations of 3${}^{\prime }$ UTR-17 bp were high in Tong sheep and Lanzhou fat-tail sheep (LFTS). Furthermore, both indel polymorphisms had significant associations with different growth characteristics (P<0.05). Among these associations, the 3${}^{\prime }$ UTR-17 bp was highly correlated with rump width in small-tail Han sheep (STHS, rams; P<0.01), and individuals with the ID genotype had better chest depth values than those with the II genotype. In this paper, two novel indels within the sheep *SIRT7* gene were identified, and genetic diversity and its effects on body size traits were explored. These findings will potentially provide useful DNA markers for the improvement of economic traits in sheep genetic breeding.

## 1 Introduction

Silent information regulator 2 (sirtuins2, SIRT2) proteins, which belonging to the class III nicotinamide adenine dinucleotide (NAD)-dependent histone deacetylases and ADP ribosyl transferases, are connected to metabolism and life span regulation in lower organisms and are highly conservative from prokaryotes to eukaryotes (O'Callaghan and Vassilopoulos, 2017). In particular, the mammalian sirtuin proteins have been reported to participate in the regulation of energy metabolism (e.g., sugar and lipid metabolism) (Li and Kazgan, 2011; Ye et al., 2017) and stress (Blank and Grummt, 2017), as well as the maintenance of genomic stability (Bosch-Presegué and Vaquero, 2014), tumor development (Roth and Chen, 2014), cell proliferation, senescence, and apoptosis (O'Callaghan and Vassilopoulos, 2017).

**Figure 1:**
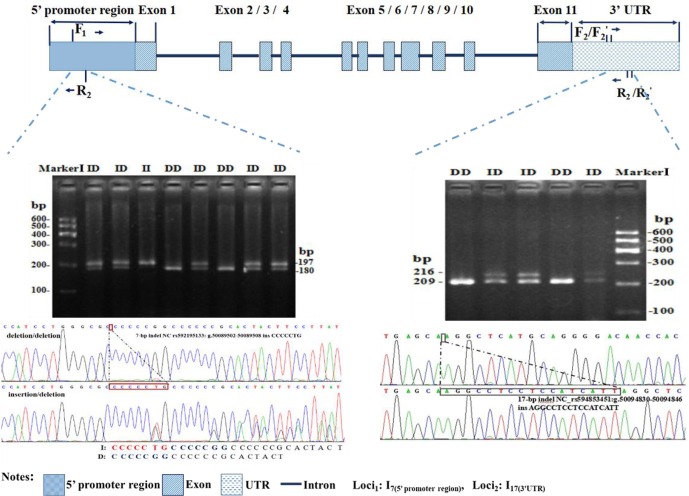
Electrophoresis diagram and sequence of 3${}^{\prime }$ UTR-insertion-17 bp loci and 5${}^{\prime }$ promoter region-insertion-7 bp within the ovine *SIRT7* gene.

Recently, *SIRT* genes have been shown to regulate the differentiation of adipocytes and myoblasts (Cioffi et al., 2015). Additionally, an increasing number of studies have revealed that the genetic diversity of *SIRT* genes is related to the economic traits of livestock. For instance, single nucleotide polymorphisms (SNPs) of the bovine *SIRT1* gene have been shown to be significantly correlated with carcass traits (such as dressing percentage, meat percentage, and carcass weight) of Chinese Luxi cattle (Liu et al., 2017). Moreover, g.13915A>G within the *SIRT4* gene significantly influences body size traits (body length, chest depth, rump length, and chest circumference) in Chinese Qinchuan cattle (Gui et al., 2016). There are seven members in the mammalian sirtuin protein family (SIRT1–SIRT7) that have different subcellular localizations. As the only member that is predominantly localized to the nucleolus (Michishita et al., 2005), SIRT7 has been reported to be involved in cellular growth and metabolism. At the cellular level, SIRT7 participates in the physiological processes of the cell stress response, proliferation, apoptosis, aging, and so on (Shin et al., 2016; Blank and Grummt, 2017). At the individual level, SIRT7 can control the progress of glycolysis (Jiang et al., 2017; Ye et al., 2017) and hepatic lipid metabolism by regulating the ubiquitin–proteasome pathway (Yoshizawa et al., 2014; Tang et al., 2015; Ye et al., 2017; Yamagata and Yoshizawa, 2018). Previous studies have revealed that SIRT7 antagonizes TGF-β signaling to inhibit breast cancer cell metastasis (Tang et al., 2017) and promotes glioma proliferation and invasion via the activation of the ERK/STAT3 signaling pathway (Mu et al., 2019). Notably, two synonymous SNP mutations of bovine *SIRT7* have been confirmed to be significantly associated with the body length, hip length, back fat thickness, and chest circumference of Qinchuan cattle (Gui et al., 2016). In contrast, there are few related reports regarding ovine* SIRT7*.

**Table 1:** PCR primer sequences of the sheep *SIRT7* gene for amplification.

Locus	Primer sequences (5${}^{\prime }$-3${}^{\prime }$)	Product size (bp)	*Tm*(${}^{\circ }$C)	Region
5${}^{\prime }$ promoter region-7bp	F${}_{1}$: TCGGCTTCGCATGTGTGT	209/216	59.97	5${}^{\prime }$ promoter region
	R${}_{1}$: GAGGCCAGAAGGAAGACAGC		60.39	
3${}^{\prime }$ UTR-17bp	F${}_{2}$: CTTGCACTGCTGTGGTTGTC	180/197	59.97	3${}^{\prime }$ UTR
	R${}_{2}$: ATTGGGTTGATGCCCCTGAG		60.03	
	F${}_{2}$': CGGGACTCTTATTCAGGGGC	192/209	58.92	
	R${}_{2}$': GGTGTGACTGCCACCTCTTT		57.50	

The ovine *SIRT7* gene is located on chromosome 11, and includes 11 exons. The molecular and biological properties of the *SIRT7* gene make it a potential candidate gene to affect body size traits in animals; however, the associations between the body size traits of sheep and mutations in the *SIRT7* gene are poorly explored and researched, especially regarding the insertion/deletion (indel) variants. Compared with other molecular markers (including SNPs), indel variants have superiority in terms of detection efficiency (Yang et al., 2016). Therefore, to explore the indel polymorphisms of the ovine *SIRT7* gene, five representative sheep breeds were studied. The Sartuul sheep (SS) is a vital wool/meat dual purpose domestic sheep breed in Mongolia, whereas the Lanzhou fat-tail sheep (LFTS), the small-tail Han sheep (STHS), the Tong sheep (TS), the and Hu sheep (HS) are representative indigenous sheep breeds in China (Li et al., 2018a, b). The objectives of this study were to investigate the novel indel polymorphisms within the ovine *SIRT7* gene in the five abovementioned sheep breeds and explore the correlations of these novel indel polymorphisms with ovine body size traits. The results of this study could potentially accelerate the progress of sheep breeding.

## 2 Material and methods

### 2.1 Animal welfare statement

In this study, all experimental processing was fully consistent with the animal welfare guidelines, laws and policies of Northwest A&F University.

### 2.2 Collection of DNA samples and data

In total, 737 same-aged sheep from five different breeds were used: four indigenous Chinese breeds, STHS (n=187), TS (n=165), LFTS (n=58), and HS (n=189); and one Mongolian native breed, SS (n=138) (Li et al., 2018a, b; Ma et al., 2018a, b). Using the same standard, the body traits of all of the sheep were measured (Zhao et al., 2017), including the cannon circumference (CaC), body weight (BW), and chest depth (ChD), among others. Thereafter, related growth trait indices, such as the chest width index (ChWI), were calculated according to the method from Lan et al. (2007).

### 2.3 Construction of genomic DNA pool

Using the high salt extraction method, the sheep genomic DNA was isolated from ear tissue which was preserved in 70 % alcohol at -80 ${}^{\circ }$C (Lan et al., 2007). The samples were then assayed using a NanoDrop 1000 spectrophotometer (Thermo Scientific, USA); the concentration of all of the diluted DNA samples was 10 ng µL${}^{-1}$ (Li et al., 2018b). Additionally, 50 samples of DNA were randomly selected and mixed to detect potential indel loci within the sheep *SIRT7* gene (Yang et al., 2017).

### 2.4 Mutation loci amplification and production sequencing

On the basis of the Ensembl database (https://asia.ensembl.org/, last access: 28 March 2019), two indel loci were selected in the sheep *SIRT7* gene, one was located in the 5${}^{\prime }$ promoter region and the other was located in the 3${}^{\prime }$ UTR (Fig. 1). Referencing the gene sequence of the sheep *SIRT7* gene (GenBank no: NC_019468.2), three pairs of amplification primers were designed using the Primer-BLAST tool in the National Center for Biotechnology Information (NCBI) database (https://www.ncbi.nlm.nih.gov/tools/primer-blast/index.cgi?LINK_LOC=BlastHome, last access: 28 March 2019) (Table 1) and were synthesized by Tsingke Biotech Company (Xi'an, China).

The amplification reaction was performed in a 13 µL volume system, including 1 µL of genomic DNA (10 ng µL${}^{-1}$), 6.5 µL of 2× EasyTaq PCR SuperMix (+dye), 0.5 µL of each primer, and 4.5 µL of ddH${}_{2}$O. The PCR amplification procedure was touch-down PCR (TD-PCR) using the steps described in previous studies (Yang et al., 2016; Xu et al., 2015; Li et al., 2018a). The amplification products were detected using 3.5 % agarose gel electrophoresis; the amplification products of each pair of primers were only sequenced if they were different (Zhang et al., 2015; Cui et al., 2018).

**Table 2:** Genetic diversity parameters of novel polymorphisms of the ovine *SIRT7* gene.

Locus	Breeds	Sizes	Genotypic frequencies			Allelic frequencies		HWE	Population parameters			
								P values				
			DD	ID	II	D	I		Ho	He	Ne	PIC
5${}^{\prime }$ promoter	LFTS	57	0.123	0.877	0.000	0.561	0.439	P<0.05	0.508	0.492	1.970	0.371
region-7bp	STHS	173	0.485	0.503	0.012	0.737	0.263	P<0.05	0.612	0.388	1.633	0.313
	TS	158	0.595	0.405	0.000	0.797	0.203	P<0.05	0.677	0.323	1.477	0.323
	HS	135	0.593	0.407	0.000	0.796	0.204	P<0.05	0.676	0.324	1.480	0.324
	SS	123	0.463	0.520	0.016	0.724	0.276	P<0.05	0.600	0.400	1.667	0.400
3${}^{\prime }$ UTR-17bp	LFTS	58	0.120	0.690	0.190	0.466	0.534	P<0.05	0.502	0.498	1.991	0.374
	STHS	187	0.278	0.529	0.193	0.543	0.457	P>0.05	0.504	0.496	1.986	0.373
	TS	165	0.261	0.400	0.339	0.461	0.539	P<0.05	0.503	0.497	1.988	0.497
	HS	189	0.238	0.656	0.106	0.566	0.434	P<0.05	0.509	0.491	1.966	0.371
	SS	138	0.297	0.507	0.196	0.551	0.449	P>0.05	0.505	0.495	1.980	0.372

Note: HWE – Hardy–Weinberg equilibrium; Ho – homozygosity; He – heterozygosity; Ne – effective allele numbers; PIC – polymorphism information content.

**Table 3:** Linkage disequilibrium test (D' and $r^{2}$) of two pairs of alleles in the different sheep breeds.

Breeds	D' test		$r^{2}$test	
LFTS		3${}^{\prime }$ UTR-17 bp		3${}^{\prime }$ UTR-17 bp
	5${}^{\prime }$ promoter region-7 bp	0.830	5${}^{\prime }$ promoter region-7 bp	0.478
STHS		3${}^{\prime }$ UTR-17 bp		3${}^{\prime }$ UTR-17 bp
	5${}^{\prime }$ promoter region-7 bp	0.914	5${}^{\prime }$ promoter region-7 bp	0.375
TS		3${}^{\prime }$ UTR-17 bp		3${}^{\prime }$ UTR-17 bp
	5${}^{\prime }$ promoter region-7 bp	0.622	5${}^{\prime }$ promoter region-7 bp	0.082
HS		3${}^{\prime }$ UTR-17 bp		3${}^{\prime }$ UTR-17 bp
	5${}^{\prime }$ promoter region-7 bp	0.999	5${}^{\prime }$ promoter region-7 bp	0.213
SS		3${}^{\prime }$ UTR-17 bp		3${}^{\prime }$ UTR-17 bp
	5${}^{\prime }$ promoter region-7 bp	0.632	5${}^{\prime }$ promoter region-7 bp	0.202

### 2.5 Statistical analysis

The mutation sequences of the two new indel loci were verified by contrasting and analyzing the sequences using BioEdit software (BioEdit, USA). Frequencies of the genotypes and alleles of the sheep *SIRT7* gene between different breeds were directly calculated using a Chi-square ($\chi^{2})$ test. Based on the GDIcall Online Calculator (http://www.msrcall.com/Gdicall.aspx, last access: 28 March 2019), the polymorphism information content (PIC) was calculated (Li et al., 2018a). In addition, the values of the $r^{2}$ test and D' test were also calculated using the SHEsis program (http://analysis.bio-x.cn, last access: 28 March 2019) to analyze the linkage disequilibrium of the populations of the two mutation loci (Shi et al., 2005; Li et al., 2009, 2018a; Wang et al., 2017). Furthermore, using an analysis of variance (ANOVA) and independent samples t tests in SPSS software (version 23.0; IBM Corp, USA), the relationships between the indels of *SIRT7* and the body size traits (e.g., chest width) in different breeds were determined (Li et al., 2018b). The genotypes were considered to be the independent variables and the growth traits were the dependent variables (Li et al., 2018a). If the data did not show homogeneity of variances and a normal distribution, a non-parametric (Kruskal–Wallis) test was used (Cui et al., 2018; Wang et al., 2019). The results were regarded as statistically significant when P<0.05, and all statistical tests were bilateral.

**Figure 2:**
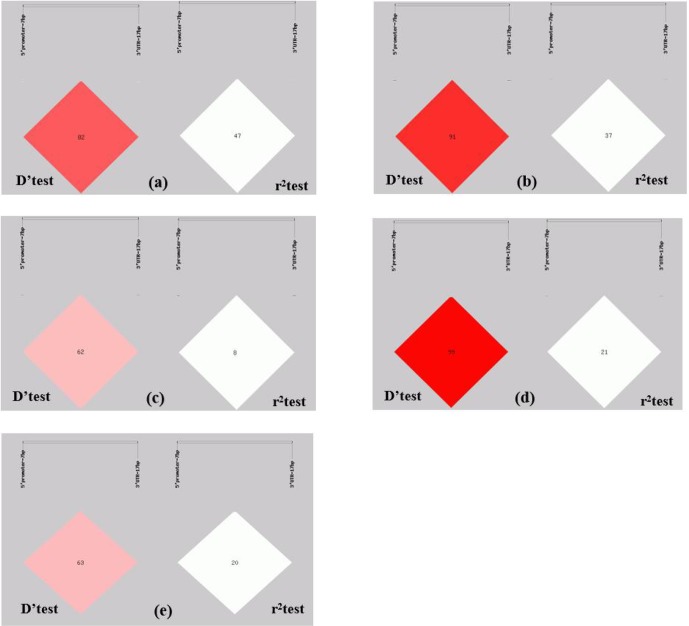
Linkage equilibrium test of two pairs of alleles within the ovine *SIRT7* gene in different populations: **(a)** Lanzhou fat-tail sheep, **(b)** small-tail Han sheep, **(c)** Tong sheep, **(d)** Hu sheep, and **(e)** Sartuul sheep. Note: the 5${}^{\prime }$ promoter region-insertion-7 bp (loci1) and 3${}^{\prime }$ UTR-insertion-17 bp loci (loci2) were chosen for haplotype analysis.

## 3 Results

### 3.1 Genotyping of individuals

Two novel indel loci within the ovine *SIRT7* gene, namely the 5${}^{\prime }$ promoter region-insertion-7 bp (5${}^{\prime }$ promoter region-7 bp) and 3${}^{\prime }$ UTR-insertion-17 bp (3${}^{\prime }$ UTR-17 bp), were genotyped and identified via gel agarose electrophoresis (3.5 %) and DNA sequencing (Fig. 1).

Polymorphisms of each indel had three different genotypes. For 5${}^{\prime }$ promoter region-7 bp (Fig. 1), genotype II (insertion/insertion) showed one band of 209 bp, genotype DD (deletion/deletion) exhibited one band of 216 bp, whereas the heterozygote genotype ID (insertion/deletion) exhibited two bands (216 and 209 bp). For 3${}^{\prime }$ UTR-17 bp (Fig. 1), three different bands were also exhibited: 180 bp, 197 bp, and 197/180 bp. After contrasting and analyzing mutant sequences, the two new indel loci were verified. The insertion sequences were CCCCCTG for 5${}^{\prime }$ promoter region-7 bp and AGGCCTCCTCCATCATT for 3${}^{\prime }$ UTR-17 bp, which were in agreement with the information in the Ensembl database.

### 3.2 Genetic parameters calculation

In Table 2, the frequencies of population parameters, genotypes, and alleles for the two indel loci in the five breeds tested are shown. For 5${}^{\prime }$ promoter region-7 bp, the DD and ID genotypes had higher frequencies than the II genotype in all of the breeds analyzed, and the D allele had a higher frequency than the I allele. For 3${}^{\prime }$ UTR-17 bp, the five breeds had a different dominant allelic frequency. In STHS, HS, and SS, the D allele had a higher frequency than the I allele, whereas the opposite was found in the LFTS and TS breeds. Additionally, the results of the population parameters demonstrated that these two indel markers displayed moderate polymorphism, and the PIC among them ranged from 0.313 to 0.497 in all of the breeds studied. Moreover, the 5${}^{\prime }$ promoter region-7 bp locus in all breeds tested, and the 3${}^{\prime }$ UTR-17  bp locus in the LFTS, TS, and HS breeds, did not conform to the Hardy–Weinberg equilibrium (HWE; P<0.05).

**Figure 3:**
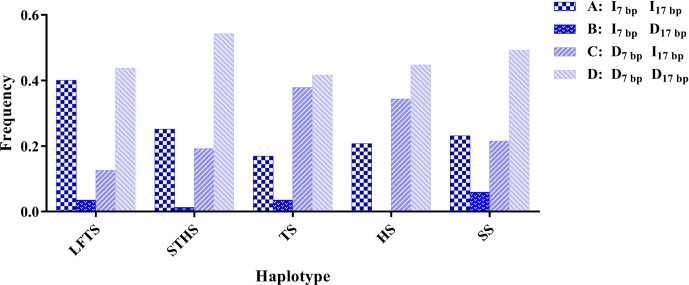
Haplotype frequency of the 5${}^{\prime }$ promoter region-insertion-7 bp and 3${}^{\prime }$ UTR-insertion-17 bp loci of the ovine *SIRT7* gene in different breeds. Note: LFTS – Lanzhou fat-tail sheep; STHS – small-tail Han sheep; TS – Tong sheep; HS – Hu sheep; and SS – Sartuul sheep.

The $r^{2}$ test showed that the 5${}^{\prime }$ promoter region-7 bp and the 3${}^{\prime }$ UTR-17 bp loci were at linkage equilibrium in all of the sheep groups tested (Table 3, Fig. 2). However, the D' test showed the opposite: in the SS and TS breeds, the D' test values showed that the two indel loci were in the weak linkage disequilibrium. Furthermore, the haplotype analysis revealed that there were four haplotypes and that “$D_{5^{\prime }\;\mathrm{promoter}\;\mathrm{region}-7\;\mathrm{bp}}\;D_{3^{\prime }\;\mathrm{UTR}-17\;\mathrm{bp}}$” had the highest incidence in all of the sheep groups detected (Fig. 3).

**Table 4:** Association of the novel indel of the ovine *SIRT7* gene and growth traits in different breeds (LSM${}^{a}$ ± SE).

Locus	Breeds	Sizes	Growth traits	Observed genotypes (LSM${}^{a}$ ± SE)			P values
				II (n)	ID (n)	DD (n)	
5${}^{\prime }$promoter	STHS (ram)	87	ChWI	83.21${}^{a}$ ± 8.21 (3)	66.47${}^{b}$ ± 1.93 (41)	68.64${}^{\mathrm{ab}}$ ± 1.34 (43)	0.035
region-7 bp	STHS (ewe)	86	CaC (cm)	–	7.08 ± 0.12 (45)	6.70 ± 0.12 (41)	0.032
			CaCI	–	11.43 ± 0.25 (45)	10.61 ± 0.20 (41)	0.013
	TS (ram)	24	BL (cm)	–	68.18 ± 1.46 (8)	73.47 ± 0.62 (16)	0.001
	HS (ewe)	135	RW(cm)	–	17.89 ± 0.11 (55)	17.38 ± 0.10 (80)	0.001
3${}^{\prime }$ UTR-17 bp	LFTS (ewe)	25	BH (cm)	71.17${}^{b}$ ± 3.11 (9)	77.48${}^{a}$ ± 1.42 (26)	68.33${}^{b}$ ± 3.18 (5)	0.028
			ChWI	68.45${}^{a}$ ± 2.93 (9)	67.65${}^{a}$ ± 2.34 (26)	55.27${}^{b}$ ± 6.14 (5)	0.035
	STHS (ram)	96	ChD (cm)	26.20${}^{c}$ ± 0.69 (16)	27.85${}^{a}$ ± 0.26 (51)	27.49${}^{\mathrm{ac}}$ ± 0.41 (29)	0.009
	STHS (ewe)	91	CaCI	10.61${}^{b}$ ± 0.31 (20)	11.44${}^{a}$ ± 0.23 (48)	10.71${}^{\mathrm{ab}}$ ± 0.27 (23)	0.039
	TS (ewe)	44	MFW (cm)	12.32${}^{b}$ ± 0.13 (16)	12.77${}^{a}$ ± 0.12 (22)	12.68${}^{\mathrm{ab}}$ ± 0.16 (16)	0.049

Note: ChWI – chest width index; CaC – cannon circumference; CaCI – cannon circumference index; BL– body length; RW – rump width; BH – body height; ChD – chest depth; and MFW – maximum forehead width. The different letters (${}^{a}$ and ${}^{b}$, or ${}^{a}$ and ${}^{c}$) beside values within the same row signify significance at the P<0.05 and P<0.01 level, respectively.

### 3.3 Association of the indel polymorphisms and body size traits

The correlation of the two abovementioned novel indels and sheep body size traits were investigated to explore whether the polymorphisms of the ovine *SIRT7* gene were related to sheep growth (Table 4). As shown in Table 4, both indel loci displayed significant relationships with the different sheep body size traits. The polymorphisms of the 5${}^{\prime }$ promoter region-7 bp were significantly associated with the chest width index (ChWI) in STHS (rams), the cannon circumference and cannon circumference index (CaC and CaCI, respectively) in STHS (ewes), the body length and chest depth (BL and ChD, respectively) in TS (rams), and the rump width in HS (ewes). Furthermore, mutations of the 3${}^{\prime }$ UTR-17 bp showed a significant relationship with the body height and ChWI in LFTS (ewes), the ChD in STHS (rams), the CaCI in STHS (ewes), and the maximum forehead width in TS (ewes). In particular, in STHS (rams), the effects of the 3${}^{\prime }$ UTR-17 bp on the chest depth was highly significant (P=0.009).

## 4 Discussion

Studies of *SIRT* genes tend to focus on the regulation of the physiological processes of cell stress responses, proliferation, apoptosis, aging, metabolism, and especially on the development of tumors (Li and Kazgan, 2011; Roth and Chen, 2014; O'Callaghan and Vassilopoulos., 2017; Ye et al., 2017). Although, in recent years, *SIRT* genes have also been shown to regulate the differentiation of adipocytes and myoblasts (Cioffi et al., 2015). Furthermore, the genetic mutations of *SIRT* genes have revealed their effects on the production traits of livestock (Gui et al., 2016; Liu et al., 2017). However, existing studies on the polymorphism of *SIRT* genes have mostly focused on SNP or haplotype mutations in cattle, whereas the study of polymorphisms of ovine *SIRT* genes is lacking. Thus, this study is the first to identify genetic diversities in the ovine *SIRT7* gene and explore the effects of these novel indel polymorphisms in representative Chinese and Mongolian sheep breeds. In this paper, two novel indels, both located in the untranslated region of the ovine *SIRT7* gene, were identified. For the 5${}^{\prime }$ promoter region-7 bp, the D allele had a higher frequency than the mutant allele (I), whereas the sheep breeds tested had different dominant allelic frequencies in 3${}^{\prime }$ UTR-17 bp. Moreover, the indel polymorphisms detected did not conform to the HWE in several of the breeds tested (e.g., 5${}^{\prime }$ promoter region-7 bp in STHS and 3${}^{\prime }$ UTR-17 bp in LFTS). Migration, crossbreeding, small sample numbers, and long-term artificial selection are suspected to be the factors contributing to the deviation from the HWE (Li et al., 2018a).

In mammals, polymorphisms of the *SIRT7* gene are involved in human longevity (Donlon et al., 2018), high-altitude adaptation (Y. Li et al., 2014), and Parkinson's disease (Jesús et al., 2013). Because of the key role of *SIRT7* in regulating lipid metabolism (Yoshizawa et al., 2014; Tang et al., 2015), glucose metabolism (Jiang et al., 2017; Ye et al., 2017), and cell growth or differentiation, the relationship between the polymorphisms tested and sheep growth traits were also analyzed. Through the association analysis, all indel loci detected were found to be significantly associated with sheep body size traits, and several associations were highly significant. For instance, in 3${}^{\prime }$ UTR-17 bp, STHS rams with the ID genotype had a greater ChD than individuals with the II genotype.

Previous studies have revealed that the mutation polymorphisms in the UTR could affect the expression of the gene by affecting the functions of elements of DNA or via microRNA-mediated post-transcriptional regulation mechanisms (Krawczak et al., 1992; Komar, 2007; G. Li et al., 2014; Tak and Farnham, 2015). Nevertheless, in this study, the location of 3${}^{\prime }$ UTR-17 bp was approximately 100 bp upstream of the seed sequence of microRNA. Furthermore, the interactions between the target gene and its adjacent genes were also speculated to contribute to this association (Li et al., 2018a, b). *MAF bZIP transcription factor G* (*MAFG*), situated in the upstream 3 kb of *SIRT7* was considered to be a transcriptional repressor whose over expression could inhibit the synthesis and metabolism of bile acid (de Aguiar Vallim et al., 2015; Katsuoka and Yamamoto, 2016). Moreover, *Phosphate cytidylyltransferase 2, ethanolamine* (*PCYT2*), located downstream 1 kb of *SIRT7*, could regulate muscle cell differentiation (Zhu et al., 2009). The mutation of UTR in the ovine *SIRT7* gene may influence the expression of the adjacent genes, thereby affecting the growth traits of sheep. However, the specific regulatory mechanisms still require further study.

In summary, two novel indel loci within the ovine *SIRT7* gene, 5${}^{\prime }$ promoter region-insertion-7 bp and 3${}^{\prime }$ UTR-insertion-17 bp, were detected in sheep. Moreover, the results of correlation analyses demonstrated that the indel loci were significantly associated with sheep growth traits, which indicated that the novel indel loci could potentially be useful DNA markers for the genetic improvement of economic traits in sheep breeding.

## Data Availability

Data sets are available upon request from the corresponding authors.
